# Bitumen and asphaltene derived nanoporous carbon and nickel oxide/carbon composites for supercapacitor electrodes

**DOI:** 10.1038/s41598-022-08159-3

**Published:** 2022-03-08

**Authors:** Dinesh Mishra, Rufan Zhou, Md. Mehadi Hassan, Jinguang Hu, Ian Gates, Nader Mahinpey, Qingye Lu

**Affiliations:** Department of Chemical and Petroleum Engineering, University of Calgary, Calgary, AB T2N 1N4 Canada

**Keywords:** Chemistry, Materials science, Nanoscience and technology

## Abstract

Asphaltenes from bitumen are abundant resource to be transformed into carbon as promising supercapacitor electrodes, while there is a lack of understanding the impact from different fractions of bitumen and asphaltenes, as well as the presence of transition metals. Here, nanoporous carbon was synthesized from bitumen, hexane-insoluble asphaltenes and *N*,*N*-dimethylformamide (DMF)-fractionated asphaltenes by using Mg(OH)_2_ nanoplates as the template with in-situ KOH activation, and used as an supercapacitor electrode material. All of the carbon exhibited large surface area (1500–2200 m^2^ g^−1^) with a distribution of micro and mesopores except for that derived from the DMF-soluble asphaltenes. The pyrolysis of asphaltenes resulted in the formation of nickel oxide/carbon composite (NiO/C), which demonstrated high capacitance of 380 F g^−1^ at 1 A g^−1^ discharge current resulting from the pseudocapacitance of NiO and the electrochemical double layer capacitance of the carbon. The NiO/C composite obtained from the DMF-insoluble portion had low NiO content which led to lower capacitance. Meanwhile, the specific capacitance of NiO/C composite from the DMF-soluble part was lower than the unfractionated asphaltene due to the higher NiO content resulting in lower conductivity. Therefore asphaltenes derived from nickel-rich crude bitumen is suitable for the synthesis of nanoporous NiO/C composite material with high capacitance.

## Introduction

The demand for renewable energy has grown rapidly in recent years due to the rapid decline of fossil fuels and growing concerns about environmental pollution. Meanwhile the demand for sustainable and clean energy is becoming more critical owing to the emergence of various electronic devices^1–3^. Therefore, the search for next generation energy storage materials and devices is very important. Supercapacitors have received a great deal of attention from the research community as energy storage devices due to their low cost, high power density, and high efficiency^4–7^. A supercapacitor consists of two electrodes immersed in an electrolyte and separated by an ion conducting but electron insulating membrane. The mechanism of charge storage in supercapacitors can be non-faradaic (electrochemical capacitor) or faradaic (pseudocapacitor)^1^. Various carbon materials with high surface area, high conductivity, morphology, size, and pore size distribution can be synthesized at large scale. In general, pure carbon materials such as activated carbon, graphene nanosheets, nanotubes, and nanocages exhibit non-faradaic double-layer energy storage mechanism, i.e. there is no electron transfer at the electrode electrolyte interface and energy storage is electrostatic in nature^8^. Meanwhile, fast reversible redox reactions occur in faradaic pseudocapacitors during the charge–discharge process^9^. Among pseudocapacitors, transition metal oxides or transition metal hydrides are mostly used due to their high theoretical specific capacitance and fast redox reactions on their surfaces^10,11^. Noble metal oxides such as RuO_2_ and IrO_2_ have been studied as electrode materials in the past^12,13^. However, the use of noble metal oxides for supercapacitors is limited due to their high cost. Instead, the use of more abundant and cheaper transition metal oxides has been explored, which has made it feasible to design supercapacitor materials with high theoretical capacitance. For example, porous nanostructured NiO and its composites have been studied as electrodes for supercapacitor because of their low cost and high theoretical capacity^14–16^. However, NiO has poor electroconductivity and therefore low charge–discharge rate and reversibility. Taking the benefit of the higher conductivity of carbon materials and high theoretical capacitance of NiO, alternatives have been explored by combining NiO with activated carbon or carbon black^16–18^.

Bitumen is an abundant natural resource in Canada which has been widely used as raw material for petroleum products. Canada alone produced 2.8 million barrels per day of crude bitumen in 2017^19^. Unlike conventional crude oil, bitumen is rich in other elements such as nitrogen, sulphur, and heavy metals. Additionally, asphaltenes, the insoluble component obtained from partial upgrading of bitumen, are also cheap and abundant carbon-rich resource. The molecular complexity of bitumen can be reduced by fractionating it using different solvents using ASTM standards^20^. Based on this method, bitumen can be fractionated into saturates, aromatics, resins, and asphaltenes. Asphaltenes are a solubility class that is soluble in light aromatics such as benzene and toluene but is insoluble in light paraffins such as the n-pentane or heptane^21^. Recently, there has been a surge in the synthesis of novel carbon materials such as nanosheet, nanoporous carbon, etc. from fossil fuels including pitch, coal, and asphaltenes^22–25^. Bitumen and asphaltenes are rich in polycyclic aromatic hydrocarbons which can be transformed into highly ordered carbon nanostructures including nanotubes and nanosheets. By using a melamine sponge template and asphaltene extracted from crude oil as the precursor^26^, or asphaltene from coal and an in-situ sheet-structure-directing agent from urea thermal polymerization^27^, the interconnected porous carbon were derived with an electrochemical capacitance of 200 F g^−1^ at 5 mV s^−1^^26^, or the porous carbon nanosheet with a graphitized-like ribbon structure with 282.9 F g^−1^ at 100 A g^−1^^27^ in a three-electrode test, respectively. Although bitumen and asphaltenes are very promising raw materials for carbon supercapacitors, there is no detailed study relating the physical and electrochemical properties of nanocarbon obtained from different fractions of bitumen. Furthermore, due to the presence of transition metals in bitumen, it can be directly used to synthesize transition metal oxide–carbon composites (TMO/C) which are known to exhibit superior performance as supercapacitors due to high conductivity of carbonaceous material and high pseudo capacitance of TMOs^28^.

Various kinds of two-dimensional (2D) materials have been used to assist in the formation of planar carbon nanosheets. Some examples of such materials which provide a guiding surface for the formation of carbon nanostructures are montmorillonite clay, Zn(OH)_2_ nanosheets, Mg(OH)_2_ nanoplates, MoS_2_ nanosheets, amino functionalized graphene oxide, NaCl, Na_2_SiO_3_, vermiculite, etc^25,29–34^. We herein report a Mg(OH)_2_ nanoplate template guided synthesis of porous carbon nanomaterials using bitumen and asphaltene fractionated from the same bitumen and their in-situ KOH activation. Mg(OH)_2_ nanoplates were chosen as template due to its cost effectiveness, simple preparation, and overall good performance of the carbon nanostructures prepared on Mg(OH)_2_ substrate. Asphaltenes were fractionated from the bitumen by precipitation using hexane. In addition, the asphaltene obtained was further partitioned into two fractions using *N*,*N*-dimethylformamide (DMF) as the solvent. The nanoporous carbon formed presents high surface area and a distribution of micro and mesopores which results in high conductivity, specific capacitance, and retention. The asphaltene fraction obtained from nickel complexes-containing bitumen led to the formation of NiO nanoparticles upon pyrolysis. The NiO/C composite obtained from asphaltenes exhibited the highest capacitance. The specific capacitance of the NiO/C composites obtained from DMF fractionated asphaltene was also measured. Interestingly, the capacitance decreased when the asphaltenes were fractionated using DMF. There was a significant decrease in the capacitance of NiO/C composite obtained from DMF insoluble fraction of asphaltenes, which was ascribed to the lower NiO content after DMF treatment. The NiO/C composite obtained from DMF soluble fraction of asphaltenes was higher than the insoluble fraction but lower than unfractionated asphaltenes. The proposed rationale for lower capacitance than unfractionated asphaltene is the higher Ni content in DMF soluble fraction of asphaltene resulted in NiO/C composites with lower conductivity.

## Experimental

### Chemicals

Toluene (anhydrous, 99.8%), n-hexane (99%), *N*,*N*-dimethylformamide (ACS reagent, ≥ 99.8%) and hydrochloric acid (ACS reagent, 37%) were purchased from Sigma Aldrich and used as received. Magnesium chloride hexahydrate (crystalline), sodium hydroxide (pellets, 98%), and potassium hydroxide (pellets, 85%) were purchased from Fisher Scientific. Oil sand sample was provided from an Alberta oil sand company. All solutions were prepared in deionized water (resistivity ≥ 18.2 MΩ cm).

### Synthesis of nanoporous carbon

Bitumen was extracted from the Alberta oil sand sample using toluene as the solvent. Asphaltenes were obtained through precipitating the bitumen in toluene solution with hexane. Asphaltenes were further separated into DMF-insoluble and DMF-soluble fractions by being dissolved in DMF and followed by filtration. Mg(OH)_2_ was prepared by slow reaction of MgCl_2_ and NaOH solution as found in the literature^35^. The precipitated Mg(OH)_2_ was filtered and washed with DI water and dried. For the preparation of porous nanocarbon, 2 g of bitumen or asphaltenes was mixed with 4 g of Mg(OH)_2_ and 8 g of KOH. The mixture was transferred to a high temperature crucible and placed inside the tube furnace under N_2_ atmosphere (flow rate 300 mL min^−1^). The sample was heated to 300 °C at the rate of 5 °C min^−1^ in N_2_ atmosphere and kept for 30 min. Finally, the temperature was raised to 800 °C at the rate of 5 °C min^−1^ and kept at that condition for another 1 h. After the completion of the reaction, the sample was cooled to room temperature and washed with HCl followed by DI water. The nanoporous carbon samples obtained from bitumen were labeled as BCNS and the nanoporous carbon obtained from hexane precipitated asphaltenes were labeled as ACNS1. The nanoporous carbons from DMF-insoluble and DMF-soluble fractions of asphaltenes were labeled as ACNS2 and ACNS3, respectively.

### Material characterization

Nanoporous carbons were characterized by scanning electron microscope (SEM), Fourier-transform infrared spectroscopy (FTIR), surface area analysis, pore size distribution, and X-ray powder diffraction (XRD) analysis. The characteristic peaks and bands were acquired by FTIR with an ATR sampling accessory (Perkin Elmer 400 FT-IR). 32 scans were performed from 500 to 4000 cm^−1^ to acquire the FTIR spectra. The XRD spectra were obtained by using a Rigaku multiplex X-ray diffractometer with a Cu X-ray source which was operated at 40 kV voltage and 40 mA current. A Micrometric ASAP 20 surface analyzer was used to measure the surface area of the samples by using the Branauer-Emmett-Teller (BET) method (N_2_ gas adsorption–desorption). Using the same equipment, pore size distributions were calculated by Barrett-Joyner-Halenda (BJH) formalisms using desorption isotherms. All the samples were degassed for 6 h at 300 °C prior to measurements. SEM images were acquired using Quanta FEG 250 field emission scanning electron microscope. All measurements were carried out under high vacuum at either 2.5 or 5 kV.

### Electrochemical measurement

The working electrode was fabricated by mixing 90% nanoporous carbon or NiO/carbon composite and 10% PTFE in 2-propanol. The mixture was sonicated for 30 min to form a homogeneous mixture which was then loaded on a 1 × 1 cm^2^ nickel foam current collector. About 1 mg of carbon was loaded on each nickel foam. After evaporating the solvent, the nickel foam with carbon was further dried at 95 °C in an oven for 1 h. For the three-electrode test, 6 M KOH was used as the electrolyte, a Pt plate as the counter electrode, and a Ag/AgCl electrode as the reference electrode. All the electrochemical tests were performed at 25 °C.

The working electrode was tested by cyclic voltammetry (CV) and galvanostatic charge discharge using PARSTAT 4000A electrochem station. Electrochemical impedance spectra were acquired between 10^5^ and 0.01 kHz using the same instrument. From the CV curve, the specific capacitance ${C}_{sp}$ of the carbon electrode under the three-electrode system was calculated by Eq. (1):

1 $${C}_{sp}=\frac{\int Idv}{mv\Delta V}$$

where $\int Idv$ is the integrated area of the CV curve, $m$ is the mass of the electrode material, $v$ is the potential scanning rate (V s^−1^), and $\Delta V$ is the potential window of the CV.

From charge-discharge experiments, the specific capacitance ${C}_{sp}$ of the carbon electrode under the three-electrode system was determined by Eq. (2):

2 $${C}_{sp}=\frac{I\Delta t}{m\Delta V}$$

where $I$ is the applied current, $\Delta t$ is the discharge time, $m$ is the mass of the electrode material and $\Delta V$ is the potential.

## Results and discussion

**Figure 1:**
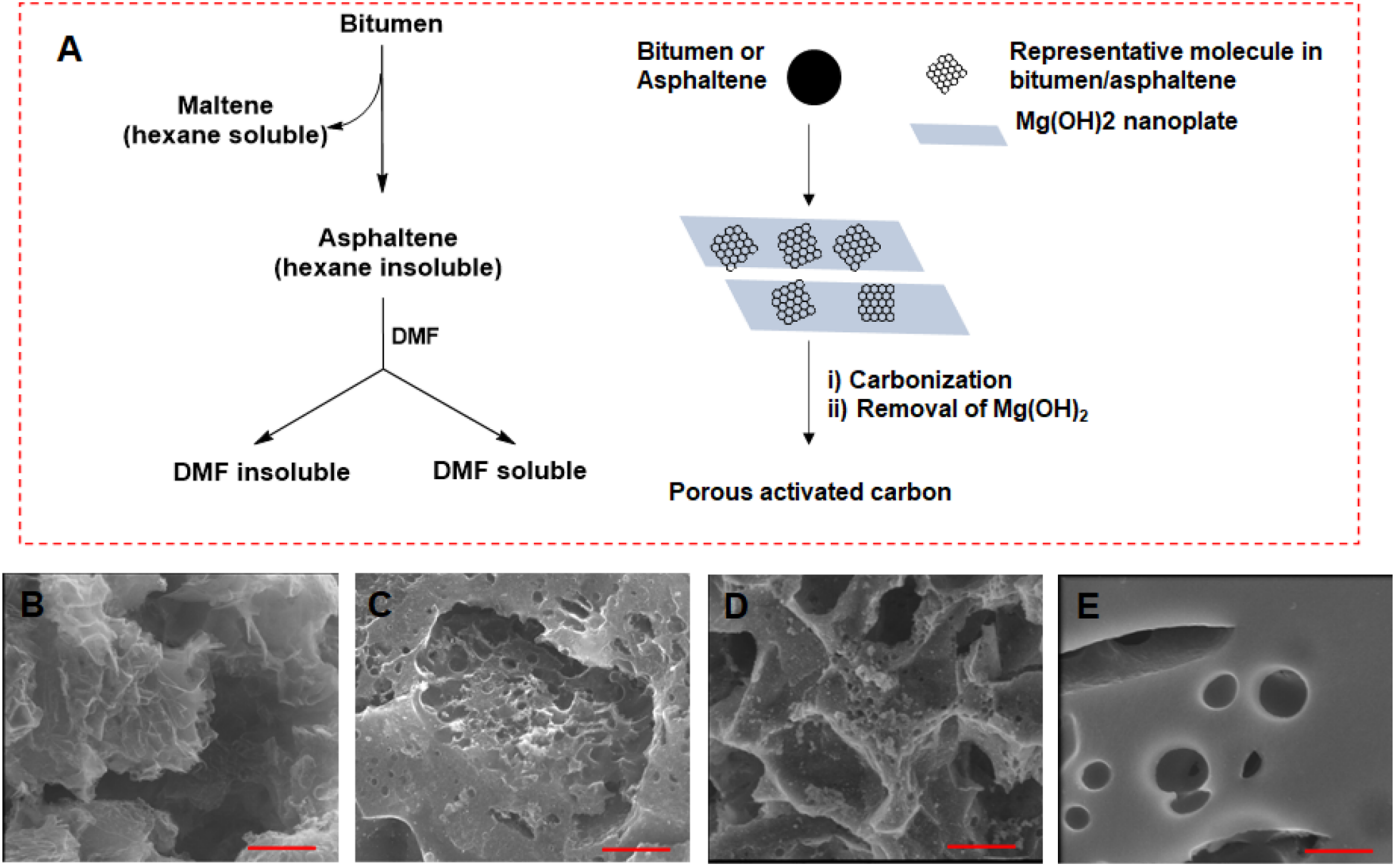
(**A**) Flow diagram representing the extraction of asphaltene from bitumen using hexane and subsequent partitioning of asphaltenes into soluble and insoluble components using N,N-dimethylformamide (DMF). SEM images of (**B**) BCNS1, (**C**) ACNS1, (**D**) ACNS2, and (**E**) ACNS3 carbon materials samples obtained from bitumen, asphaltenes, DMF-soluble and DMF-insoluble asphaltenes, respectively. The scale bar is 1 μm.

**Figure 2:**
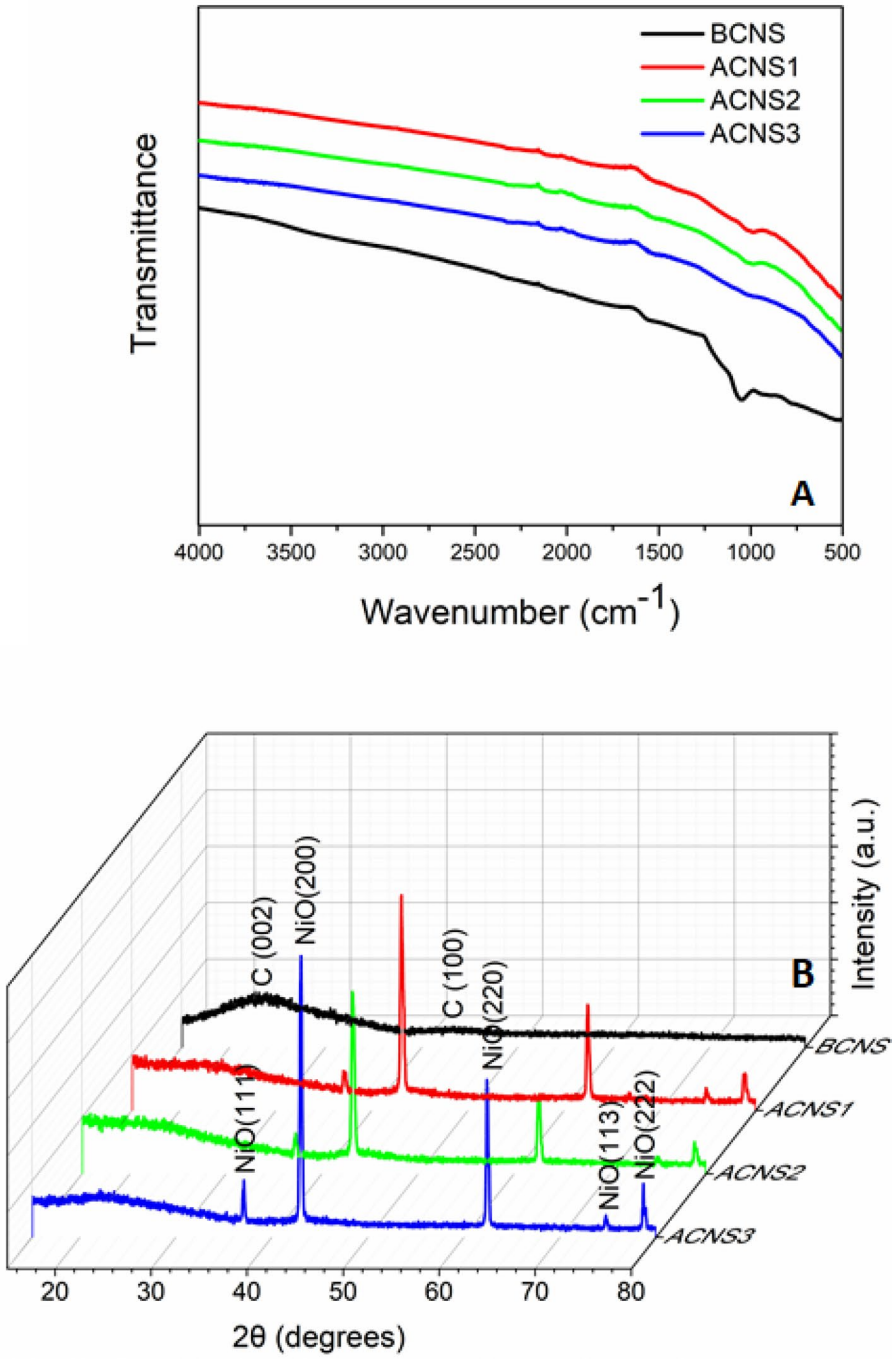
(**A**) FTIR spectra (**B**) XRD spectra of BCNS, ACNS1, ACNS2, and ACNS3.

As shown in Fig. 1A, the bitumen samples were fractionated into hexane-soluble maltenes and hexane-insoluble asphaltenes and the asphaltenes were subsequently partitioned into DMF soluble and insoluble fractions. The nanoporous carbon and NiO/C composites, i.e., BCNS1, ACNS1, ACNS2, ACNS3 obtained from bitumen, asphaltene, DMF-soluble and DMF-insoluble asphaltenes, respectively, were characterized using SEM, FTIR, XRD, BET surface area analysis, and pore size distribution analysis. The SEM images of the four samples are shown in Fig. 1B–E, respectively. As shown in Fig. 1B, BCNS sample had flaky appearance due to sheet-like structures, whereas ACNS1 and ACNS2 samples (Fig. 1C,D) looked spongy with large number of pores on the surface. Meanwhile, ACNS3 sample looked very compact with large pore sizes (Fig. 1E). This also accounts for the rather small surface area (discussed below) of ACNS3 sample compared to the others. Such significant difference of sample surface morphology structure indicates the contributions of different oil fractions in forming the carbon network. Asphaltenes contain high aromaticity and are easy to polymerize or cross link for preparing networked carbon materials or graphitic carbon structure. The existence of hexane-soluble portion probably weakens interactions among asphaltenes and helps the formation of flake carbon structures from bitumen precursor. The interactions of different portions in asphaltenes also lead to different crosslinking degree and pore sizes during the pyrolysis. The FTIR spectra of the carbon and NiO/C composites are shown in Fig. 2A. Carbon nanomaterials are good absorbers of radiation and the FTIR spectrum of these materials could include lots of noise. Weak peaks corresponding to C-O bond stretching were observed in BCNS samples but were not found in ACNS samples. This means the existence of maltene portion in bitumen precursor led to trace C–O in the final carbon structure and thus different surface properties for BCNS to ACNS samples. C–H bands were absent in both BCNS and ACNS samples. Almost featureless FTIR spectra indicate the absence of functional groups on the particle surface. This implies that active functional groups such as nitrogen and sulfur were eliminated from the carbon structure during KOH etching activation^26^. XRD spectra of BCNS and ACNS samples are shown in Fig. 2B. A broad peak around 2θ = 23° and weak peak around 2θ = 46° confirmed the presence of graphitic material in all the samples. However, in the ACNS samples, five distinct peaks corresponding to NiO were identified in addition to the broad carbon peaks, which suggests the formation of NiO/C composites in ACNS1, ACNS2, and ACNS3 samples. This also indicates the strong binding of Ni to the asphaltenes precipitated from hexane and thus Ni was not removed during the further processing of asphaltenes. Such NiO existence in carbon was found out later to be crucial for charge storage as supercapacitor electrode materials. The peak corresponding to NiO(200) plane overlapped with C(100) plane. The Scherrer equation was used to determine the crystallite size of NiO using the XRD data. The calculated crystallite sizes of NiO for ACNS1, ACNS2, ACNS3 samples were 25.7 ± 2.1 nm, 25.3 ± 1.3 nm, and 30.8 ± 2.2 nm, respectively. The fractionation by polar solvent DMF will lead to different portions of asphaltenes and content of Ni in the precursors. The increase of the size of NiO particles in the DMF extracted ACNS3 sample could be due to higher Ni content in the DMF-soluble fraction.

**Figure 3:**
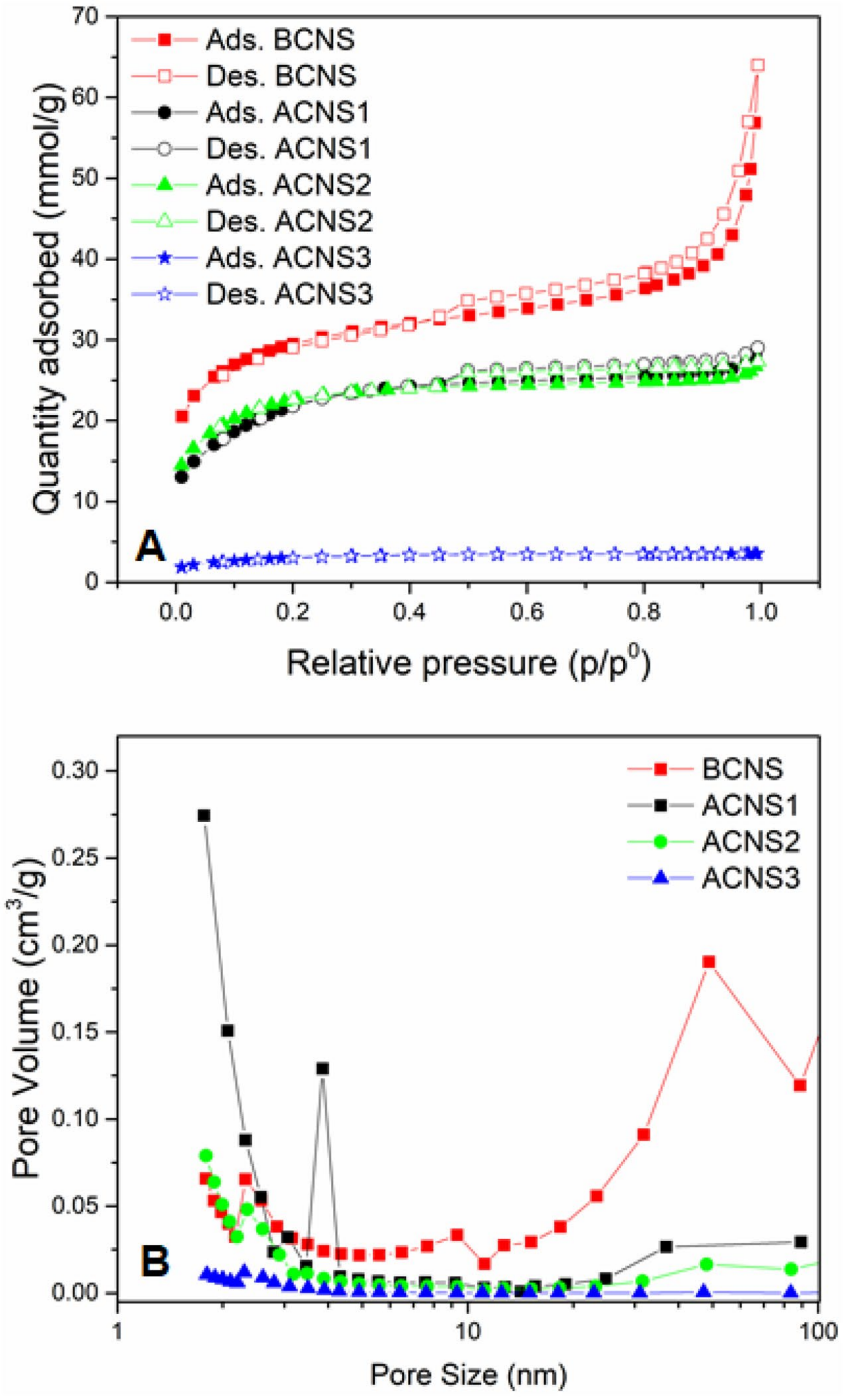
(**A**) N_2_ adsorption–desorption isotherms and (**B**) pore size distribution of BCNS, ACNS1, ACNS2, and ACNS3.

Figure 3A,B show the N_2_ adsorption–desorption isotherms and the pore size distributions of the nanoporous carbon samples. The surface area and pore volumes of the nanoporous carbon materials are listed in Table 1. The adsorption–desorption isotherms indicate there is a distribution of micro and mesopores. The hysteresis loop was seen in all samples indicating multilayer adsorption and capillary condensation in the mesoporous structure^24^. As shown in Table 1, BET surface area was 2117 m^2^ g^−1^ for BCNS, 1594 m^2^ g^−1^ for ACNS1, 1589.5 m^2^ g^−1^ for ACNS2, and 222 m^2^ g^−1^ for ACNS3. Despite the larger surface area of BCNS, the pore size distribution showed abundant mesopores in the BCNS whereas the pore size distribution was much narrower and in the micropore region for ACNS1 and ACNS2 samples. In general, abundant micropores are associated with higher specific capacitance of the carbon materials^36^. The lower surface area (222 m^2^ g^−1^) of ACNS3 sample is comparable to the surface area of Ni/C composite prepared by carbonization of Ni-phthalocyanine complexes reported in the literature^37^. Polar solvent such as DMF has been used in the past to extract organometallic complexes (e.g. vanadium or Ni-porphyrin complex) from asphaltenes^38,39^. Thus, the DMF extract is considerably rich in metallic content which results in Ni/C composite with higher Ni and lower carbon concentration upon carbonization. The lower adsorption of ACNS3 and absence of abundant pores in ACNS3 suggests that the dominant charge storage mechanism in this sample will be pseudocapacitance and not an electrochemical double layer capacitance (EDLC) mechanism such as is found in carbon rich materials.

**Table 1:** Physical properties of BCNS, ACNS1, ACNS2, and ACNS3.

Sample	SSA (m^2^ g^−1^) (p/p0 = 0.3)	BET (m^2^ g^−1^)	V_tot_ (cm^3^ g^−1^)	V<2 nm (cm^3^ g^−1^)	V>2 nm (cm^3^ g^−1^)
BCNS	2112	2117	1.1	0.20	0.9
ACNS1	1594	1641	0.67	0.29	0.38
ACNS2	1590	1614	0.53	0.20	0.33
ACNS3	222	226	0.082	0.028	0.054

**Figure 4:**
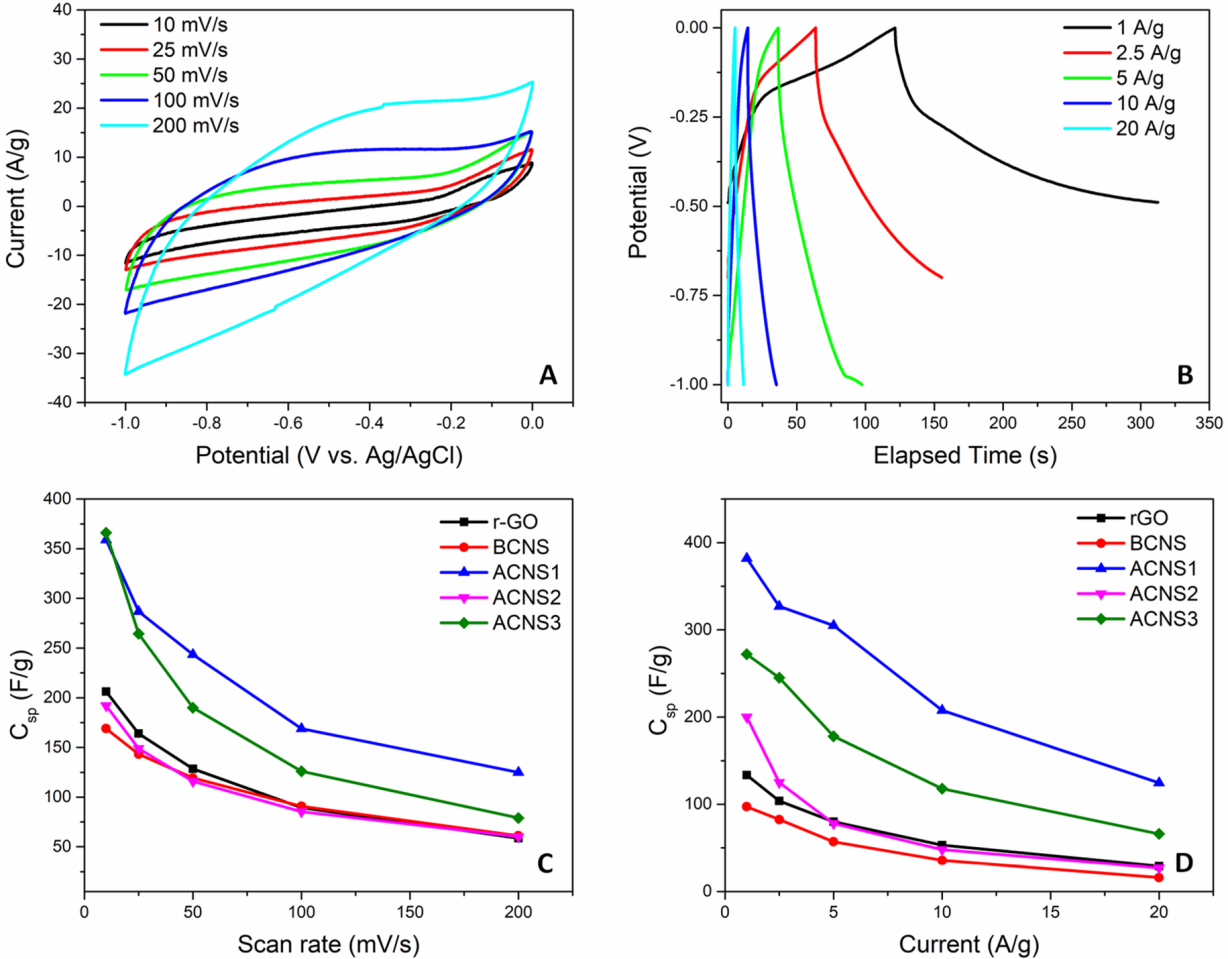
(**A**) CV and (**B**) galvanostatic charge–discharge of ANS1 (**C**) Specific capacitance vs scan rate of all CNS and reduced GO (rGO) (**D**) specific capacitance vs charge–discharge current of all CNS and rGO.

**Figure 5:**
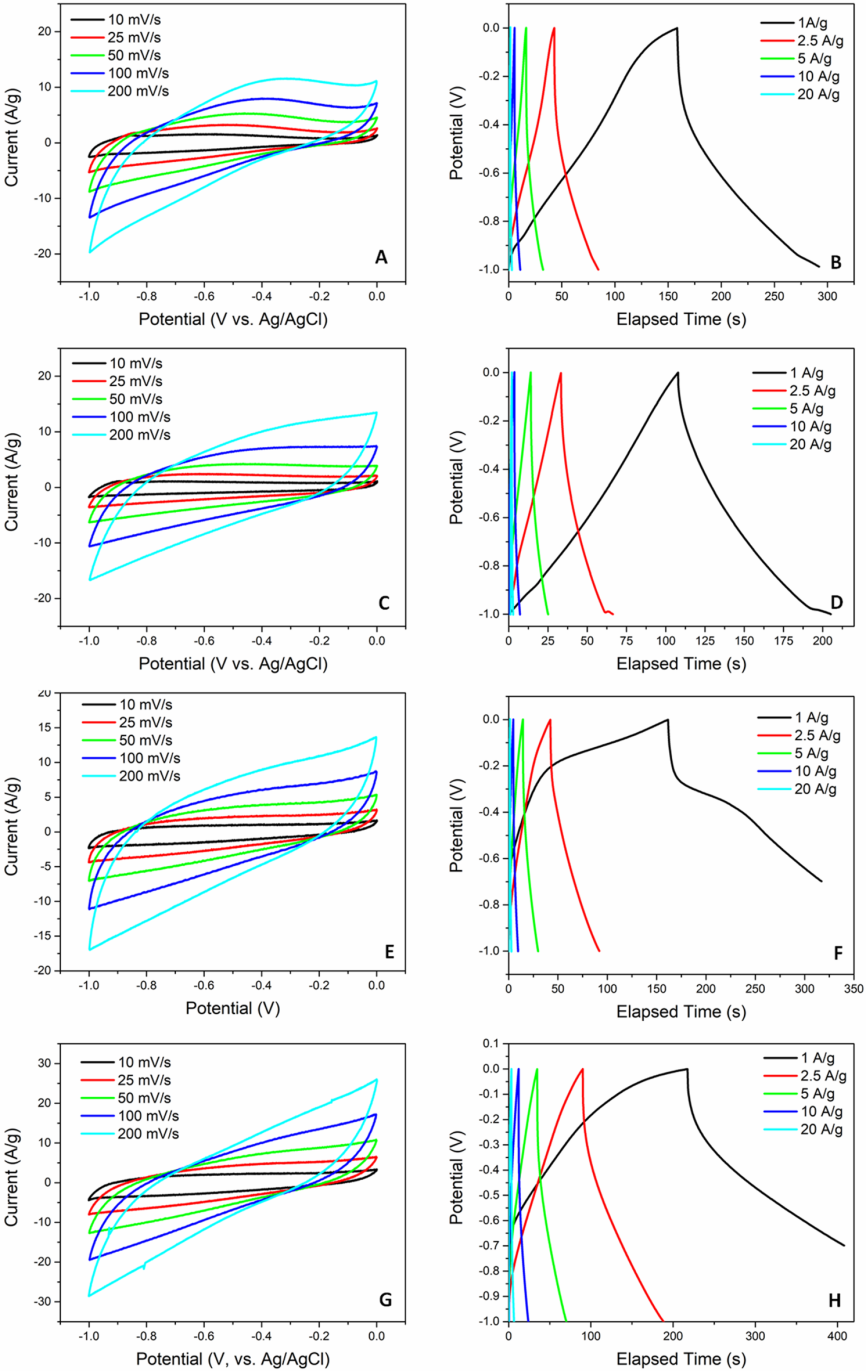
CV and galvanostatic charge discharge of (**A**,**B**) rGO, (**C**,**D**) BCNS (**E**,**F**) ACNS2, and (**G**,**H**) ACNS3, respectively.

**Figure 6:**
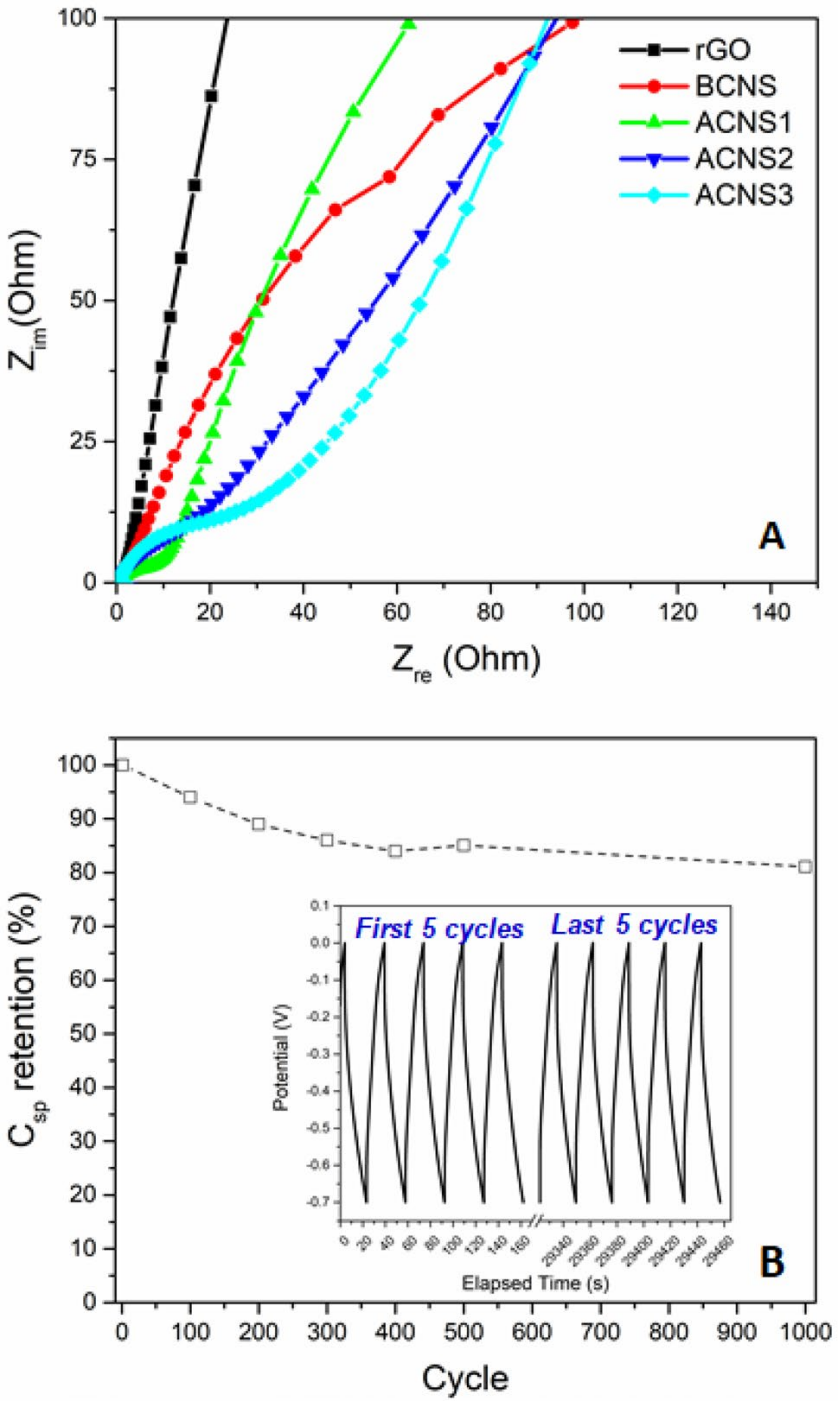
(**A**) Electrochemical impedance spectroscopy of rGO, BCNS, ACNS1, ACNS2, and ACNS3 electrodes, and (**B**) cyclic performance of ACNS1 electrode at 5 A g^−1^.

Figure 4A shows the CV curves of ACNS1 at the scan rates of 10, 25, 50, 100, and 200 mV s^−1^ over the potential range 0 to − 1 V. The large current response and quasi rectangular shape suggests reversible electrochemical double layer capacitance whereas the slight redox peaks at − 0.2 V reveals the pseudocapacitive behavior of the ACNS1 sample which is due to NiO. Galvanostatic charge–discharge of the ACNS1 at current densities 1, 2.5, 5, 10, and 20 A g^−1^ are shown in Fig. 4B. The galvanostatic charge–discharge curves deviate from symmetry, which implies that the supercapacitive behavior of ACNS1 resulted from both pseudocapacitance and EDLC. Charging–discharging times were longest at 1 A g^−1^ and decreased as the current was increased. The CV and galvanostatic charge–discharge curves of BCNS, ACNS2, and ACNS3 are shown Fig. 5. The CV curves of these electrodes were similar in shape to the ACNS1 electrode but had lower current values. The galvanostatic charge discharge curves of BCNS electrode was nearly symmetric indicating dominant EDLC phenomenon. On the other hand, the charge-discharge curves of ACNS2 and ACNS 3 were similar in shape to the ACNS1 electrode due to the pseudocapacitance along with EDLC. For comparison, the CV and galvanostatic charge–discharge curves of reduced graphene oxide (rGO) were also measured (Fig. 5). The gravimetric capacitances obtained from CV and galvanostatic charge–discharge curves of BCNS, ACNS1, ACNS2, ACNS3, and reduced GO are shown in Fig. 4C,D, respectively. The data show that ACNS1 had the highest capacitance compared to the other samples. The gravimetric capacitance of ACNS1 is 359 F g^−1^ at potential scan rate 10 mV s^−1^. ACNS3 had specific capacitance of 365 F g^−1^ at the same scan rate but it is lower than ACNS1 at higher scan rates. Similarly, gravimetric capacitance measured from galvanostatic charge–discharge measurements were highest for ACNS1 as shown in Fig. 4D. The calculated gravimetric capacitance from GCD measurement at 1 A g^−1^ was 380 F g^−1^. Capacitances of BCNS, ACNS2, and reduce GO were lower than that of ACNS1 and ACNS3. The higher specific capacitance ${C}_{sp}$ for ACNS1 and ACNS3 is due to the pseudocapacitance of NiO combined with the EDLC of carbon. For ACNS2 and ACNS3, which are prepared from the DMF insoluble and DMF soluble fractions of asphaltene, respectively, the change of the NiO/C ratios in these samples during the fractionation process led to the decrease in capacitance. The Ni content decreased in the DMF insoluble fraction which led to NiO/C composite with lower NiO content. Thus, the pseudocapacitive contribution was decreased leading to overall decrease of the ${C}_{sp}$ of ACNS2. Meanwhile, the DMF soluble fraction, which was rich in Ni, formed NiO/C composite with higher NiO content and the capacitance increased again in ACNS3. However, the capacitance was lower than ACNS1 which may be due to the increased resistance of the NiO component and was further discussed with the following impedance measurement results (Fig. 6). The effect of NiO content in NiO/C composite on the capacitance has been reported in prior studies. For example, Lota and coworkers prepared NiO-activated carbon composites with three different ratios: 34% NiO and 66% activated carbon, 17% NiO and 83% activated carbon, and 7% NiO and 93% activated carbon. Their results indicated that the low amount of NiO (7%) resulted in the highest capacitance^40^. Moreover, the smaller NiO crystal size in ACNS1 could be beneficial since it can provide higher specific surface area for charge storage. It should also be noted that most of the literature reported also apply 5–10% conductive carbon black to improve the conductivity of the electrodes^23,41^. In this work, we prepared electrodes without adding carbon black. Table 2 summarizes the capacitance of various carbon and carbon-composite materials compared to the one reported in this study, indicating very comparable performance achieved in this work.

**Table 2:** Capacitive performance of different carbon and NiO based materials.

Materials (method)	Electrode	Capacitance
Reduced graphene oxide (micropatterning GO on Au followed by autoclave to obtain rGO)^42^	Ultrathin rGO microelectrode on Au	285 F g^−1^ at 1A g^−1^ (2-electrode)
Graphene paper (mechanically pressed graphene aerogel)^43^	Active materials on titanium foam	172 F g^−1^ at 1 A g^−1^ (2-electrode)
Carbon nanosheets (self-assembly of coal based carbon dots on Mg(OH)_2_ template)^31^	Active materials, acetylene black, PTFE (85:10:5 w/w) on nickel foam	230 F g^−1^ at 1 A g^−1^ (3-electrode)
Carbon nanosheets (carbonized coal-derived asphaltene with in-situ urea polymerization as template)^27^	Active materials, PTFE and carbon black (85:5:10 w/w) on nickel foam	315 F g^−1^ at 1 A g^−1^ (3-electrode)
Porous carbon network (carbonized crude oil-derived asphaltene with a melamine sponge template^26^	Active materials, polyvinylidene fluoride (PVDF), conductive carbon (80:10:10 w/w)	200 F g^−1^ at 5 mV s^−1^ (3-electrode)
Nitrogen doped graphene films (plasma enhanced chemical vapor deposition)^44^	Active materials, PVDF on nickel foam (90:10 w/w)	282 F g^−1^ at 1 A g^−1^ (2-electrode test)
Graphene-polyaniline composite paper (electropolymerization of aniline on graphene paper)^45^	Active materials	763 F g^−1^ at 1 A g^−1^ (3-electrode)
NiO/reduced graphene oxide composite (ball-milling of graphite oxide and Ni powder)^46^	Active materials, carbon black, PTFE (70:20:10)	590 F g^−1^ at 1 A g^−1^ (3-electrode)
NiO/carbon nanofibers (calcination of Ni(OH)_2_ deposited carbon fibers)^47^	Active materials, Nafion	526 F g^−1^ at 1 A g^−1^ (3-electrode)
NiO nanoparticles in mesoporous carbon nanospheres (carbonization of silica/nickel silicate/resorcinol mixture followed by NaOH etching) ^48^	Active materials, PTFE, graphite (80:10:10 w/w) on nickel foam	406 F g^−1^ at 1 A g^−1^ (3-electrode)
NiO/C composite (vertically grown NiO nanosheets on N-doped carbon hollow spheres)^18^	Active materials, PVDF, acetylene black (80:10:10 w/w) on nickel foam	585 F g^−1^ at 1 A g^−1^ (3-electrode)
This work: asphaltene derived nano NiO/C composite (pyrolysis of bitumen-derived asphaltenes with Mg(OH)_2_ template and in-situ KOH activation)	active materials, PTFE (90:10 w/w) on nickel foam	380 F g^−1^ at 1 A g^−1^ (3-electrode)

Figure 6A shows the Nyquist plots of the BCNS, ACNS1, ACNS2, ACNS3 electrodes along with reduced GO electrode. All of the electrodes have low electrode resistance as indicated by the small value of the x-intercept. The curve for reduced GO has very steep curve at low frequency which is indicative of a good EDLC behavior. A similar trend is observed for the BCNS electrode but it has a curve with lower slope. For the ACNS electrodes, a semicircle with diameter increasing in the order ACNS1 < ACNS2 < ACNS3 is observed indicative of interfacial resistance due to NiO. The steep curve of the diffuse layer for ACNS1 is comparable to reduced GO while the slopes of the curve are lower for ACNS2 and ACNS3. To test the stability of the electrode, cycling tests were conducted galvanostatically between 0 and − 1 V. Figure 6B shows the cyclic stability of the ACNS1 electrode during the galvanostatic charge–discharge experiment at the current density of 5 A g^−1^ over 1000 cycles. As shown, the electrode retains ~ 85% capacitance after 1000 cycles thus exhibiting good cyclic stability.

## Conclusions

Nanoporous carbon and NiO/carbon composite materials were prepared directly from bitumen and asphaltenes, respectively. The as prepared carbon and NiO/carbon composite have large surface area with abundant pores for ion adsorption and transport. Bitumen derived nanoporous carbon had capacitive performance which was comparable to chemically reduced GO. The charge storage in BCNS is through the EDLC mechanism. Asphaltene derived NiO/C composite electrodes on the other hand exhibit enhanced capacitance due to the pseudocpacitive behavior of the NiO present in the nanoporous NiO/carbon composite. High theoretical capacitance of NiO due to fast reversible redox reactions along with high conductance and EDLC behaviour of activated nanoporous carbon which makes it suitable for supercapacitor electrode material. The capacitance of the asphaltene derived NiO/C electrodes without adding conductive carbon black is comparable to most of the carbon-based supercapacitors reported in the literature which also use conductive carbon for enhanced conductivity. This study suggests that asphaltene derived from crude bitumen which is rich in nickel is suitable for the synthesis of activated nanoporous NiO/C composite material with high capacitive performance and cycling stability.
